# Role of Furin in Colon Cancer Stem Cells Malignant Phenotype and Expression of LGR5 and NANOG in KRAS and BRAF-Mutated Colon Tumors

**DOI:** 10.3390/cancers14051195

**Published:** 2022-02-25

**Authors:** Jean Descarpentrie, Marcos J. Araúzo-Bravo, Zongsheng He, Alexia François, Álvaro González, Patricia Garcia-Gallastegi, Iker Badiola, Serge Evrard, Simon Pernot, John W. M. Creemers, Abdel-Majid Khatib

**Affiliations:** 1 Reprogramming tumor activitY and associaTed MicroEnvironment (RYTME), Bordeaux Institute of Oncology (BRIC)-UMR1312 Inserm, B2 Ouest, Allée Geoffroy St Hilaire CS50023, 33615 Pessac, France; jean.descarpentrie@u-bordeaux.fr (J.D.); alexia.francois@u-bordeaux.fr (A.F.); p.garciagallastegi@gmail.com (P.G.-G.); s.evrard@bordeaux.unicancer.fr (S.E.); 2 Computational Biology and Systems Biomedicine Group, Biodonostia Health Research Institute, C/Doctor Beguiristain s/n, 20014 San Sebastian, Spain; marcos.arauzo@biodonostia.org; 3 Department of Gastroenterology, Daping Hospital, Army Medical University, Chongqing 400042, China; zongsheng.he@kuleuven.be; 4 Laboratory of Biochemical Neuroendocrinology, Department of Human Genetics, KU Leuven, B-3000 Leuven, Belgium; john.creemers@kuleuven.be; 5 Department of Cell Biology and Histology, Faculty of Medicine and Nursing, University of Basque Country (UPV/EHU), 48940 Leioa, Spain; iker.badiola@ehu.eus; 6 Institut Bergonié, 33000 Bordeaux, France; s.pernot@bordeaux.unicancer.fr

**Keywords:** cancer stem cells, colon cancer, LGR5, NANOG, KRAS, BRAF, calcium

## Abstract

**Simple Summary:**

Colorectal cancer (CRC) is one of the most common malignancies in the digestive system. We have previously shown that the proprotein convertase Furin is involved in calcium regulation in cancer cells. In this study, we revealed that the malignant phenotype of colon cancer stem cells is repressed by Furin inhibition that is associated with reduced expression of LGR5 and Nanog and dysregulated expression of several calcium regulators involved in colon cancer. Our data support the idea that targeting Furin in colorectal cancer stem cells may constitute a potential therapeutic approach.

**Abstract:**

Proprotein convertases or PCs are known to regulate the malignant phenotype of colon cancer cells by different mechanisms, but their effects on cancer stem cells (CSCs) have been less widely investigated. Here, we report that PCs expression is altered in colon CSCs, and the inhibition of their activity reduced colon CSCs growth, survival, and invasion in three-dimensional spheroid cultures. In vivo, repression of PCs activity by the general PC inhibitors α1-PDX, Spn4A, or decanoyl-RVKR-chloromethylketone (CMK) significantly reduced tumor expression levels of the stem cell markers LGR5 and NANOG that are associated with reduced tumor xenografts. Further analysis revealed that reduced tumor growth mediated by specific silencing of the convertase Furin in KRAS or BRAF mutated-induced colon tumors was associated with reduced expression of LGR5 and NANOG compared to wild-type KRAS and BRAF tumors. Analysis of various calcium regulator molecules revealed that while the calcium-transporting ATPase 4 (ATP2B4) is downregulated in all the Furin-silenced colon cancer cells, the Ca^2+^-mobilizing P2Y receptors, was specifically repressed in BRAF mutated cells and ORAI1 and CACNA1H in KRAS mutated cells. Taken together, our findings indicate that PCs play an important role in the malignant phenotype of colon CSCs and stem cell markers’ expression and highlight PCs repression, particularly of Furin, to target colon tumors with KRAS or BRAF mutation.

## 1. Introduction

Colorectal cancer (CRC) is the second leading cause of mortality among cancer patients in the world and is the third most diagnosed cancer globally [1]. No single cause for CRC has been identified but it results from the cumulative effects of multiple and sequential genetic alterations. These include mutations of the tumor suppressor gene adenomatous polyposis coli (APC) and the proto-oncogene KRAS, allowing the activation of Wnt/β-catenin and Ras/ERK pathways, respectively [2,3,4]. Found in up to 90% of CRC patients, APC gene inactivating mutations promote the initiation of CRC [5], whereas KRAS gene mutations have been detected in approximately 40% of CRC patients, seemed to occur during all stages of CRC [6,7] and were found to mediate colon cancer initiation through CSCs activation [7].

Although it is well known that the tumor malignancies are caused by the induction of CSCs originating from a small subpopulation of tumor cells, the mechanism of action and factors involved in their promotion and properties in colon cancer are poorly understood [8,9]. CSCs are involved not only in tumor progression but have also been linked to metastasis induction and relapse after chemotherapy, the major causes of lethality in various cancer patients [10]. Previously, CSCs were reported to derive from oncogenic reprogramming of normal stem cells, where various stemness factors, such as NANOG and LGR5, which play a key role are expressed. Altered expression of these stemness factors was found to mediate CSCs malignant phenotype acquisition and tumor progression [11,12,13]. Indeed, LGR5 (also known as GPR49) is identified as a marker of both colon normal stem cells and CSCs [14]. This G-protein-coupled receptor is the main target of Wnt signaling [15] and was found to be upregulated in all phases of cancer cell transformation, and it remains upregulated after malignant phenotype acquisition [16]. Furthermore, the analysis of LGR5 expression in CRC patients revealed that its high expression significantly correlates with resistance to 5-fluorouracil (5-Fu) treatment [17]. Similarly, NANOG, a differentiated homeobox (HOX) domain protein initially identified as an embryonic stem cells molecule with self-renewal and multipotent transcriptional regulatory functions [18], was also found to mediate CSCs’ stemness, their invasiveness, and metastasis, as well as resistance to cancer treatment. NANOG overexpression in colon CSCs was found to promote tumorigenicity in preclinical models [19] and its inhibition was found to be able to attenuate colon CSCs properties and to enhance sensitization to therapy [20,21]. Accordingly, while NANOG is silenced in normal cells, its abnormal expression has been reported in various human cancers, and was associated with poor prognosis and lower survival rate in colon cancer patients [19,21,22,23]. NANOG overexpression in colorectal CSCs was found to promote tumorigenicity in preclinical models [19].

Previously, a comparative transcriptomic study in stem- and non-stem-cancer cells identified an enrichment of calcium regulator genes in CSCs. The CSCs showed enhanced sensitivity to calcium homeostasis and signaling [24], indicating a key role for calcium in CSCs’ malignant phenotype. The calcium ion is a ubiquitous second messenger, involved in the regulation of a wide range of cellular processes, including cell proliferation, migration, and death [25]. Many extracellular signals from the microenvironment that mediate Ca^2+^ mobilization are also involved in the malignant phenotype of CSCs. These include various growth factors, and receptors such as VEGF, TGF-β, IGF-IR, and Notch are synthesized as inactive precursor proteins that are converted to their bioactive forms by one or more of the proprotein convertases (PCs) [26,27,28,29].

To date, seven PCs have been identified: namely, Furin, PC1, PC2, PC4, PACE4, PC5, and PC7. These enzymes are members of the subtilisin/kexin family implicated in the conversion of a large number of secretory proteins. The latter, synthesized as larger proteins, are cleaved at the motif (K/R/H)-(X)n-(K/R), where n= 0, 2, 4 or 6 and X any amino acid except Cys [26,27,28,29,30]. The most promising protein-based specific inhibitors of PCs are the individual PC-pro-segment-based inhibitors, the serpin variant α1-PDX and the serpin Spn4A [26,27,28,29,30]. Previously, the inhibition of the convertases in CSCs was associated with upregulated expression of various metallothioneins known as tumor suppressor genes, of which the loss in colon cancer patients was associated with bad prognosis [31]. On the other hand, Furin was reported to interfere with calcium mobilization [29,32], and PCs repression reduced the malignant phenotype of cancer cells and resistance to apoptotic agents [29]. As a result, in recent years, the concept of CSCs has interesting implications in the exploration of new and effective therapies that target the altered signaling pathways of CSCs. In this study we evaluate the importance of the convertase’s activity on the malignant phenotype of colon CSCs. The effect of PCs repression on the expression of the stemness markers LGR5 and NANOG and various calcium regulators and channels in KRAS and BRAF mutated cells are also investigated.

## 2. Materials and Methods

### 2.1. Cell Culture and Formation of SPHEROIDS Mimicking CSCs

The characteristics and origin of the control CT-26 cells and the same cells stably expressing α1-PDX [33], and Spn4A [29] and control HT-29, DLD1 and HCA7 cells and the same cells with silenced Furin (KO) [34], were described previously. Both SW620 and SW480 cell lines were obtained from the American Type Culture Collection (ATCC, Manassas, VA, USA). Cells were routinely maintained in Dulbecco’s minimal essential medium (DMEM) (Gibco, Saint Aubin, France) supplemented with 10% heat-inactivated fetal bovine serum (FBS), 1% sodium pyruvate, 0.1 mg/mL streptomycin, 100 U/mL penicillin at 37 °C in a 5% CO2-humidified atmosphere. For SW480, SW620, CT26, HT-29 tumorsphere formation mimicking CSCs phenotype, as previously described [32], cells were cultured in DMEM/F-12 supplemented with N2, B27 supplements (Thermo Fisher Scientific, Waltham, MA, USA), and growth factors [20 ng/mL basic fibroblast growth factor (bFGF) and 20 ng/mL epidermal growth factor (EGF)] (Sigma, St. Louis, MO, USA) in a 96-well round bottom plate that facilitated the production of homogeneous spheroids of cells.

### 2.2. RNA Extraction and Quantitative Real-Time PCR

To compare the expression levels of PCs and CSC markers in parental and CSCS and during tumor progression, total RNA was isolated from the colon cancer cell lines and mice-developed tumors using Trizol solution (Molecular Research Center, Cincinnati, OH, USA), according to the manufacturer’s protocol. The RNA was reverse transcribed using the high-capacity cDNA reverse transcription kit (Applied Biosystems, Thermo Fisher Scientific, Courtaboeuf Cedex, France) and used for real-time PCR. Real-time PCR was performed using power SYBR Green PCR Master Mix (Applied Biosystems, France), as previously described [33] with primers indicated in Supplementary Table S1. The quantitative polymerase chain reaction (qPCR) data were acquired with the StepOnePlusTM Real-Time PCR System (Applied Biosystems, France). The expression levels were normalized to β-Actin housekeeping gene. Gene expression was assessed using the comparative threshold cycle (Ct) method.

### 2.3. Spheroid Growth and Collagen Invasion Assays

To quantify the cell proliferation rate of the CSCs, spheroids were generated and cultured in a 96-well round bottom plate and equal-sized spheroids were used to assess PCs inhibition effects. Images of CSCs cultured in individual chambers were acquired from Day 0 to Day 4 with an inverted microscope (Nikon). The surface area of each CSC spheroid was measured using the Fiji Macro image analysis program [35]. For CSCs invasion assay, spheroids were mixed with Type I Collagen (Corning, NY, USA) and incubated separately in each well plate in the presence or absence of the PC inhibitors. All the images were captured with the same setting parameters and invasion of collagen type I was measured by the deduction of the total area from the central area, using the Fiji Macro analysis program [35].

### 2.4. Proprotein Convertases Activity Assay

The universal PCs substrate, the fluorogenic peptide pERTKR-MCA, was used to evaluate the PCs activity in cell lysates, as previously described [33,36]. In brief, tissue or cell extracts were incubated with pERTKR-MCA (100 µM) during the indicated time periods in the presence of 25 mM Tris, (pH 7.4), 25 mM methyl-ethane-sulfonic acid, and 2.5 mM CaCl_2_, at 37 °C, and the fluorometric measurements were performed using a spectrofluorometer (FLUOstar OPTIMA; BMG Labtech, Cs/Marne, France).

### 2.5. Cell Viability Assay

The cell viability was evaluated using the MTT assay, as previously described [37]. Control and α1-PDX-expressing cells or CMK-treated cells were incubated with the chemotherapeutic agent 5-fluorouracil (5-FU) for 24 h and were immersed with an MTT solution (0.5 mg/mL) for 3 h at 37 °C. Then absorbance was measured at 570 nm using the ELISA plate reader.

### 2.6. Mouse Model

All research animals were housed in the University of Bordeaux in a temperature-controlled environment. All experimental procedures were approved by the Institutional Animal Care and Use Committee (University of Bordeaux) and were conducted under the supervision of a trained veterinarian. Male 4- to 6-week-old nu/nu mice were inoculated subcutaneously in the right flank with 1 × 10^6^ control colon carcinoma cells HT-29, CT-26 or the same cells expressing the PC inhibitors α1-PDX or Spn4A. Tumor formation was monitored every 2–3 days, and mice were sacrificed at the end of the experiments. Tumor volume was calculated as previously described [33].

### 2.7. Statistical Analysis

Unless otherwise indicated, Student’s t test was used to determine the statistical significance of differences between the means of several experiments. A probability value less than 0.05 was considered to be statistically significant. Data are shown as mean ± SEM or mean ± SD.

## 3. Results

### 3.1. Enrichment of CSCs after Sphere-Forming Is Associated with Altered Proprotein Convertases (PCs) Expression

**Figure 1:**
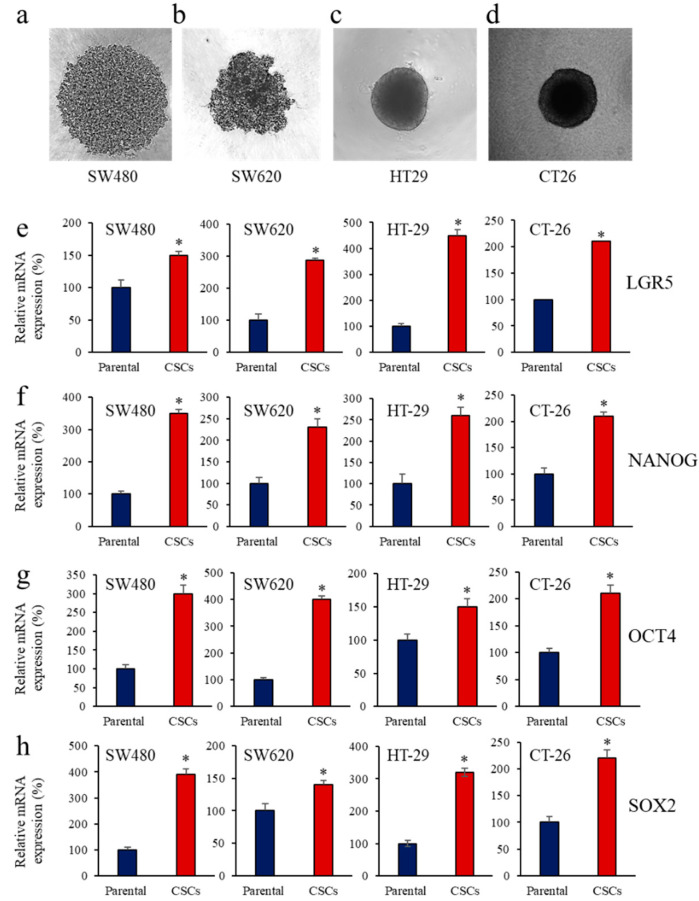
Expression of CSC markers during enrichment of colon CSCs. (**a**–**d**), morphology of CSC mimicking spheres generated from parental colon cancer cells SW480 (**a**), SW620 (**b**), HT-29, (**c**) and CT-26 (**d**). (**e**–**h**), Total RNA was extracted from parental cells and CSCs and analyzed by real-time PCR using specific primers for LGR5 (**e**), NANOG (**f**), OCT4 (**g**) and SOX2 (**h**) or β-actin under the conditions described in the text. Shown are the quantification of mRNA expression relative to control parental cells assigned 100%. Results are representative of three experiments. Data are mean ± SEM (n = 3). * *p* < 0.005.

The sphere-forming assay has been widely used to enrich CSCs in vitro. Thus, to determine whether PCs expression in cancer stem cells (CSCs) is altered, spheres were generated from the parental colon cancer cells SW480 (Figure 1a), SW620 (Figure 1b), HT-29, (Figure 1c), and CT-26 (Figure 1d). The mRNA expression of the CSC markers, LGR5 (Figure 1e) and NANOG (Figure 1f), were significantly induced in the spheres of the colon cancer cell lines SW480, SW620, CT-26 and HT-29, compared to their parental cells. The function of these CSC markers is also mediated with the assistance of OCT4, SOX2, and other complexes. Analysis of OCT4 and SOX2 expression revealed their upregulated levels (Figure 1g,h) in the SW480, SW620, CT-26, and HT-29 colon CSCs.

**Figure 2:**
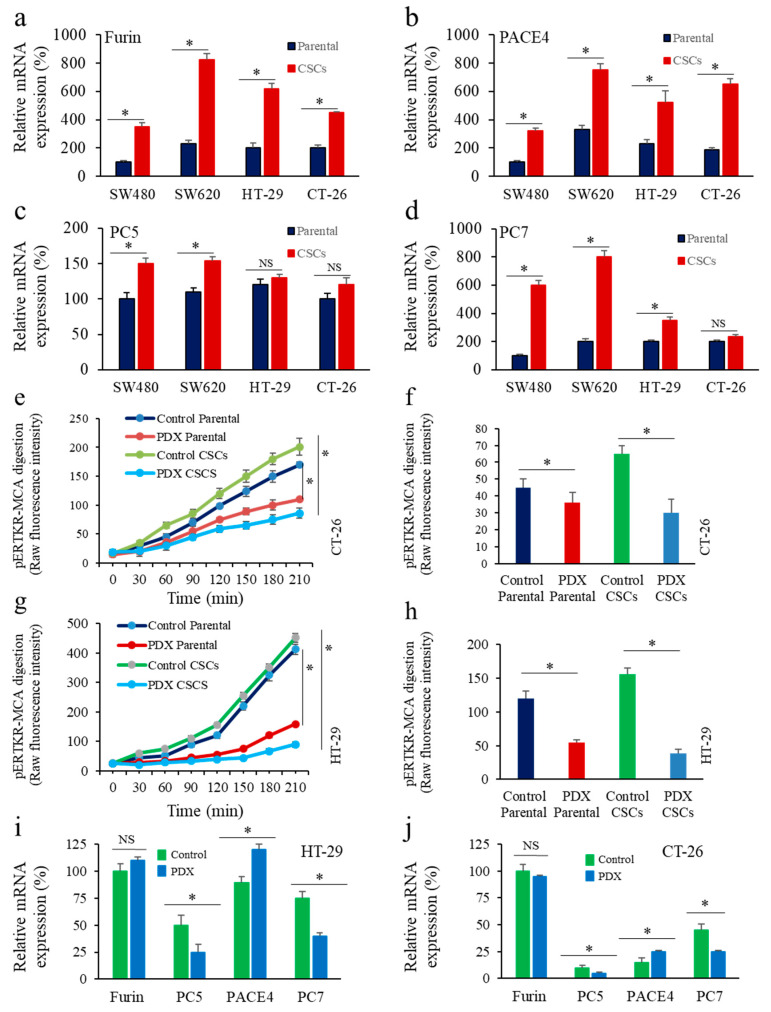
Proprotein convertases (PCs) expression and activity inhibition during enrichment of colon CSCs. (**a**–**d**), real time PCR analysis of Furin (**a**), PACE4 (**b**), PC5 (**c**) and PC7 (**d**) in colon CSCs and their parental cells in the colon cancer cells SW480, SW620, HT-29, and CT-26. (**e**–**h**), Effect of the PCs inhibitor α1-PDX (PDX) expression on the indicated parental colon cancer cells and corresponding CSCs was assessed by evaluating cells’ ability to digest the fluorogenic peptide pERTKR-MCA. (**i**,**j**), Effect of α1-PDX on the expression of Furin, PC5, PACE4 and PC7 in HT-29 (**i**) and CT-26 (**j**) cells. Results shown in the bar graphs represent PCs activity after 30–90 min of incubation and evaluated as raw fluorescence intensity (RFI). Results are representative of three experiments and data are mean ± S.E.M performed in triplicate. * *p* < 0.05. NS: not significant.

We next compared the expression of all the PCs found in the secretory pathway, namely Furin, PACE4, PC5 and PC7 in these colon CSCs and their parental cells. Thus, analysis of sphere-forming subgroups revealed up-regulated expression of Furin (Figure 2a), PACE4 (Figure 2b) and PC7 (Figure 2d). Only marginal changes were observed in PC5 expression in these cells (Figure 2c). These data suggest the potential implication of PCs in the tumoral properties of CSCs.

### 3.2. Inhibition of PCs Activity and CSC Gene Expression

To determine if CSCs in colon cancer involve PCs activity for their malignant phenotype, we first inhibited the PCs activity in cancer cells following expression of the general PC inhibitor α1-PDX [33]. As illustrated in Figure 2e–h, expression of α1-PDX (PDX) in the colon cancer cells HT-29 and CT-26 significantly affects their PCs activity. The PCs activity inhibition was observed in the parental and corresponding sphere-forming subgroups (Figure 2e–h). Analysis of PCs expression in the absence and presence of α1-PDX revealed that this inhibitor had no effect on Furin expression but reduced PC5 and PC7 expression, and enhanced PACE4 expression in both cell lines (Figure 2i,j).

**Figure 3:**
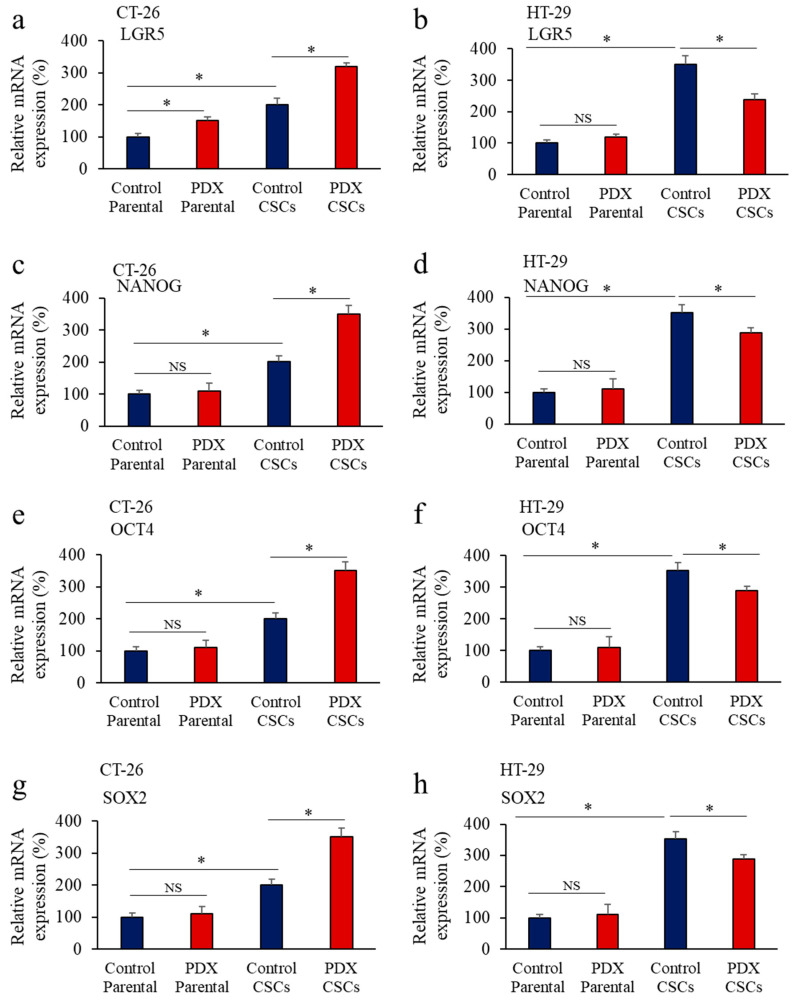
Proprotein convertases (PCs) activity repression and CSC gene expression in colon CSCs. Effect of the PCs inhibitor α1-PDX expression on the expression of LGR5 (**a**,**b**), NANOG (**c**,**d**), OCT4 (**e**,**f**) and SOX2 (**g**,**h**) in the indicated parental colon cancer cells and their corresponding CSCs was assessed by real-time PCR analysis. Results are representative of three experiments and data are mean ± S.E.M performed in triplicate. * *p* < 0.05. NS: not significant.

We next analyzed in colon cancer cells the expression of LGR5, NANOG, OCT-4, and SOX2 in the presence and absence of α1-PDX. As indicated, the expression of LGR5 (Figure 3a,b), NANOG (Figure 3c,d), OCT-4 (Figure 3e,f) and SOX2 (Figure 3g,h) was increased in CSCs compared to parental cells. In CT-26 and HT-29 CSCs, the expression of these markers was increased and reduced, respectively, in the presence of α1-PDX (Figure 3a–h). These findings, suggest that the difference expression levels of the PCs found in HT-29 and CT-29 (Figure 2i,j), and the potential specific role of each PC in the expression of LGR5, NANOG, OCT-4, and SOX2 are affected differently by α1-PDX. These results suggest that PCs activity may be involved in the CSCs malignant phenotype and can be affected by PC inhibitors.

### 3.3. PCs Inhibition Promotes CSCs Proliferation and Invasion Blockade

**Figure 4:**
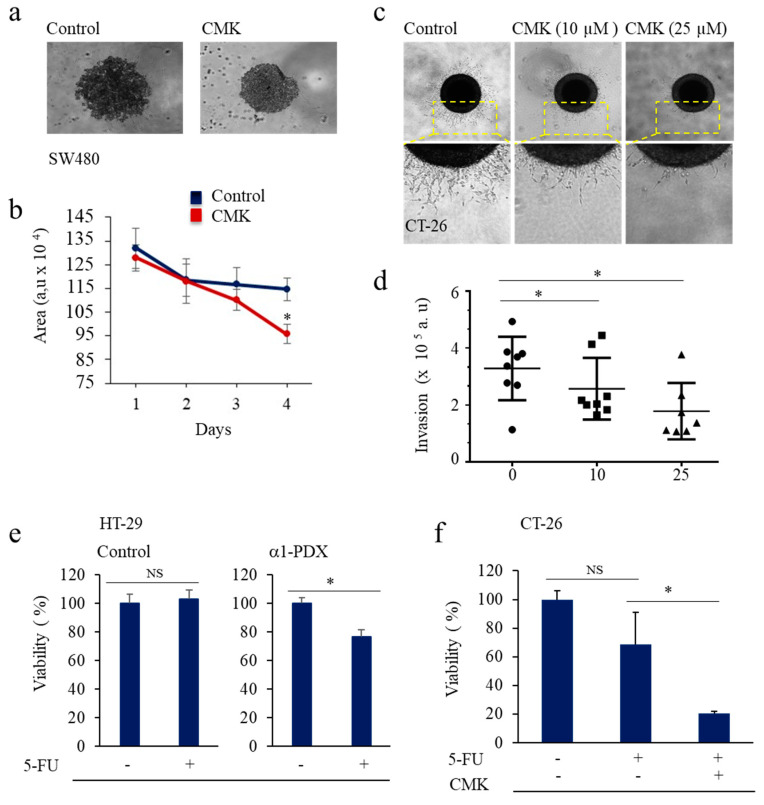
Proprotein convertases inhibition mediates CSC malignant phenotype repression. (**a**), representative image of control and CSCs-CMK treated cells. (**b**), CSCs generated from SW480 cells were incubated with the synthetic PCs inhibitor, the decanoyl-RVKR-chloromethylketone (CMK), for the indicated time periods. Results shown in the bar graph represent the growth of the CSCs that was evaluated by the measurement of the total area of the spheroids, using the Fiji Macro analysis program. (**c**) Representative image of control and CSCs-CMK treated cells invasion. (**d**) CSCs spheroids were mixed with Type I Collagen and incubated in the presence or absence of CMK. Invasion of collagen type I was measured by the deduction of the total area from the central area, using the Fiji Macro analysis program. (**e**,**f**) Effect of 5-FU (50 μM) on the viability of CSC HT-29 and CSC CT-26 in the absence and presence of PCs inhibitors α1-PDX and CMK, as assessed by MTT assay and calculated with respect to control (100%). Results are representative of three experiments and data are mean ± S.E.M performed in triplicate. * *p* < 0.05. NS: not significant.

To investigate whether PCs activity influences CSCs proliferation, spheres were first generated from SW480 and SW620 cells and were incubated with the synthetic general PCs inhibitor, the decanoyl-RVKR-chloromethylketone (CMK). We found that CMK reduced the growth of the colon cancer spheres compared to control cells (Figure 4a,b). Similarly, to investigate the invasion of colon cancer spheroids into extracellular matrix (ECM) that may be present in the perivascular niche, spheroids generated from the highly invasive colon cancer cells CT-26 were embedded into type-1 collagen (Figure 4c,d) and after 1 day in culture, spheres were treated with indicated concentrations of CMK. We found that control tumor cells had invaded the collagen using single cell and collective cell migration modes, suggesting that these tumor cells exhibit bi-modal forms of invasion. In the presence of CMK, both the single cell and collective cell migration modes were repressed in a dose-dependent manner (Figure 4c,d). These results suggest a direct link between the growth and invasion of colon CSCs and PCs activity.

### 3.4. Inhibition of PCs Activity Increases Chemoresistance to 5-FU

The most commonly used drug in the clinical treatment of CRC today is 5-Flourouracil (5-FU) [38]; however, resistance to this treatment is common, especially in the metastatic setting. Thereby, we investigated the effect of PCs repression on the viability of the colon CSCs HT-29 and CT-26 in the absence and presence of 5-FU (50 μM) (Figure 4e,f). We found that while incubation of control CSCs with 5-FU at this concentration, for 24 h had no effect on cell viability, these CSCs became significantly sensitive towards 5-FU in the presence of α1-PDX or CMK compared to controls.

### 3.5. Inhbition of PCs in Colon Cancer Cells Reprsses LGR5 and NANOG Expression in Mice-Induced Tumors

**Figure 5:**
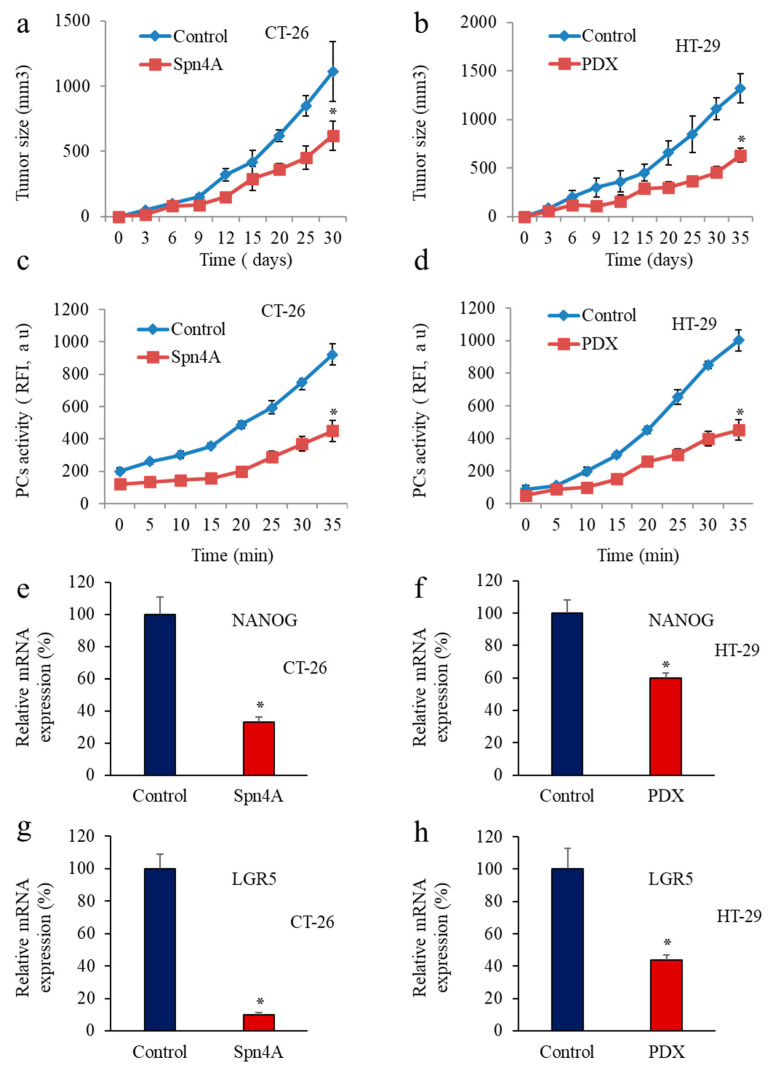
Inhibition of tumor growth by Spn4A and α1-PDX is associated with reduced PCs activity and repressed LGR5 and NANOG expression. (**a**,**b**), Control colon cancer cells and the same cells stably expressing Spn4A (Spn4A) or α1-PDX (PDX) were injected subcutaneously into mice. The animals were monitored for tumor formation every 2–3 days. Results are representative of three experiments. Values are mean ± S.E.M (n = 6 per group). * *p* < 0.05; (**c**,**d**), Subcutaneously developed tumors were removed and their protein extracts were incubated with pERTKR-MCA. Substrate cleavage was evaluated as raw fluorescence intensity (RFI) at indicated time periods. (**e**,**g**), Results shown in the bar graph represent NANOG (**e**,**f**) and LGR5 (**g**,**h**) expression in the developed tumors derived from control and Spn4A-expressing tumor cells or α1-PDX-expressing cells analyzed by real-time PCR. Results are representative of three experiments and data are mean ± S.E.M performed in triplicate. * *p* < 0.05.

Since CSC markers, LGR5 and NANOG have been shown to be progressively expressed during carcinogenesis and promote cancer cell proliferation and tumor formation [22,39], we next evaluated the impact of PCs repression in tumor cells on LGR5 and NANOG expression during tumor progression. For this, we used the colon carcinoma cells CT-26 and HT-29 and the same cells that express the PCs inhibitors Spn4A and α1-PDX. As previously observed [30,33], inhibition of the PCs in these cells reduced their ability to induce tumor growth in nude mice (Figure 5a,b), confirming the importance of the PCs in the malignant phenotype of tumor cells. To verify the reduced PCs activity in the developed tumors, PCs activity was analyzed by assessing the ability of tumor-derived protein extracts to digest the fluorogenic peptide pERTKR-MCA using vitro enzymatic digestion assay. The results in Figure 5c,d revealed that the extent of cleavage of pERTKR-MCA by protein extracts of HT-29 and CT-26-derived tumors was higher than that of HT-29 and CT-26-expressing PC inhibitors cells-derived tumors, revealing the inhibition of the PCs activity in these cancer cells-derived tumors. Analysis of LGR5 and NANOG expression levels by RT-PCR in these tumors revealed that in mice inoculated with tumor cells expressing PC inhibitors, the level of NANOG was reduced by up to 60% and 40% in Spn4A and α1-PDX-expressing cells injected mice, respectively (Figure 5e,f). Similarly, analysis of LGR5 expression in these tumors revealed its reduced expression by up to 80% and 60% in the presence of Spn4A and α1-PDX, respectively (Figure 5g,h). These findings directly linked the PC’s activity to LGR5 and NANOG expression during tumor progression.

### 3.6. Furin Silencing Mediates Repression of LGR5 and NANOG in Mice-Induced Tumors with KRAS or BRAF Mutation

**Figure 6:**
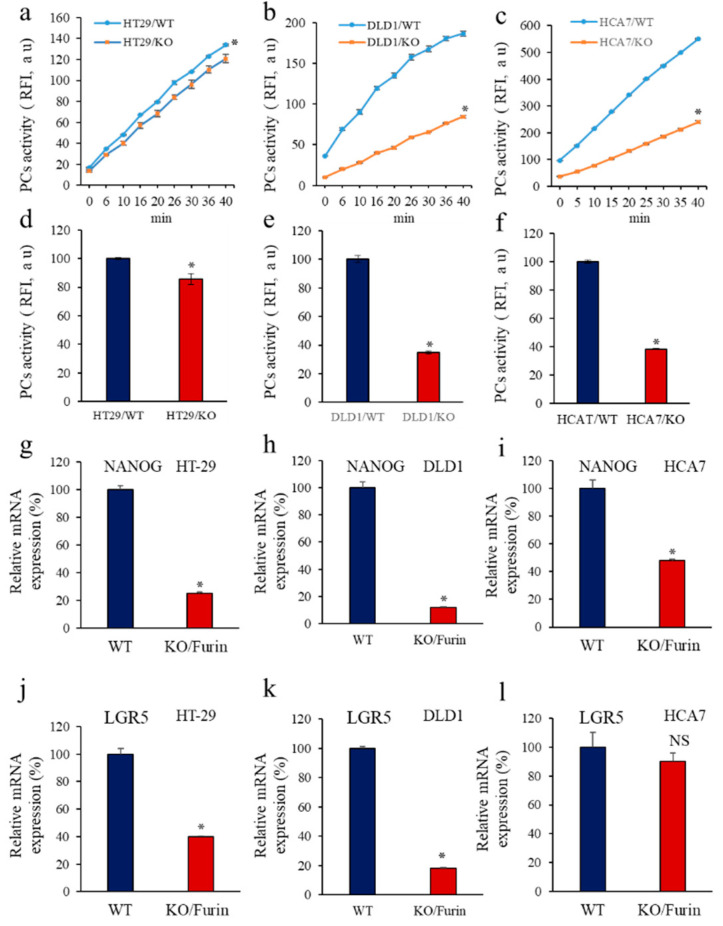
Furin silencing mediates repression of LGR5 and NANOG in KRAS or BRAF mutated tumors. (**a**–**c**), PCs activity was analyzed by assessing the ability of the colon cancer cell lines DLD1 with KRAS mutation, HT29 with BRAF mutation and HCA7 with WT KRAS and BRAF with silenced (KO) Furin to digest the fluorogenic peptide pERTKR-MCA in the in vitro enzymatic digestion assay. (**d**,**f**), Results shown in the bar graph represent PCs activity after 20 min of incubation. (**e**),Furin silencing mediates repression of LGR5 and NANOG in KRAS or BRAF mutated tumors. (**g**–**l**), Results shown in the bar graph represent NANOG (**g**–**i**) and LGR5 (**j**–**l**) expression analyzed by real-time PCR in the developed tumors derived from control DLD1, HT29 and HCA7 and the same cells with silenced Furin. Results are representative of three experiments and data are mean ± S.E.M performed in triplicate. * *p* < 0.05. NS: not significant.

Previously, Furin inactivation was found to impair the malignant phenotype of colon cancer cells with activating KRAS or BRAF mutations but not with wild-type (WT) KRAS or BRAF [34]. In vivo, while the control cancer cells induced tumor formation in nude mice, silencing of Furin (KO) reduced the ability of cells with KRAS or BRAF mutation to induce tumor growth [34]. In contrast, repression of Furin in the wild-type HCA7 cells shows a marginal effect compared with control cells [34]. Interestingly, in colon CSCs, mutation of KRAS or BRAF was involved in their malignant characteristics [7]. Therefore, to evaluate the importance of Furin in the expression of LGR5 and NANOG during tumor progression mediated by colorectal cancer cells harboring these mutations, we used the colon cancer cell lines DLD1 with KRAS mutation, HT29 with BRAF mutation, and HCA7 with WT KRAS and BRAF all with genetically silenced Furin gene (CRISPR/Cas9 approach) [34]. Analysis of PCs activity in DLD1, HT29, and HCA7 showed reduced PCs enzymatic activity, as assessed by their inhibited ability to digest pERTKR-MCA (Figure 6a–f). Analysis of NANOG (Figure 6g–i) and LGR5 (Figure 6j–l) expression in the derived mice tumors revealed that NANOG was downregulated in HT29/BRAF and DLD1/KRAS KO cells-derived tumors as well as in HCA7/KO cells-derived tumors by up to 70%, 80%, and 50%, respectively (Figure 6g–l). In contrast, while no significant effect on LGR5 expression was observed in HCA7/KO cells-derived tumors, LGR5 expression was reduced by up to 60% and 80% in HT29/BRAF and DLD1/KRAS KO cells-derived tumors, respectively (Figure 6j–l). These results indicate that the reduced tumor growth mediated by Furin silencing in colon cancer with mutated KRAS or BRAF is associated with reduced LGR5 and NANOG expression. In HCA7 cells lacking these mutations, the effect of Furin repression on LGR5 was not significant (Figure 6l) and had less effect on NANOG expression (Figure 6i).

### 3.7. Calcium Regulators’ Expression in Cancer Cells with Repressed Furin and KRAS or BRAF Mutation

**Figure 7:**
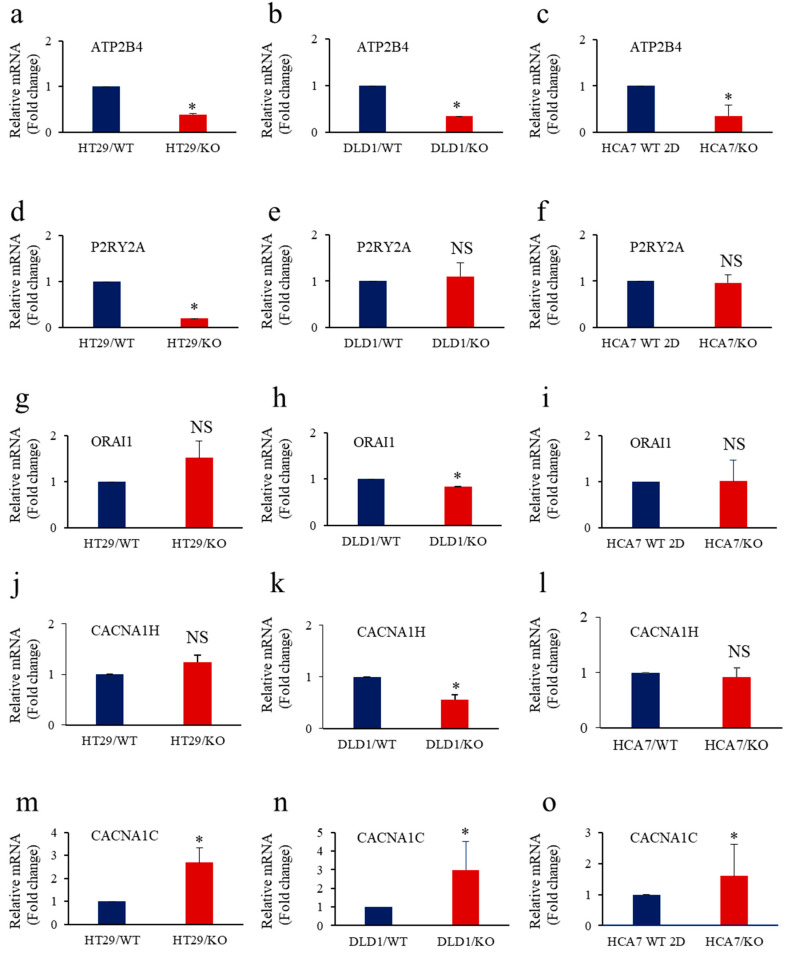
Calcium regulators’ expression in KRAS or BRAF mutated cells lacking Furin. (**a**–**o**), Total RNA was extracted from the control colon cancer cell lines DLD1 (KRAS mutation), HT29 (BRAF mutation) and HCA7 (WT KRAS and BRAF) and the same cells with silenced (KO) Furin and analyzed by real-time PCR using specific primers for ATP2B4 (**a**–**c**), P2RY2A (**d**–**f**), ORAI1 (**g**–**i**), CACNA1H (**j**–**l**), CACNA1C (**m**–**o**), and β-actin under the conditions described in the text. Shown are the quantification of mRNA expression relative to control parental cells assigned 1. Results are representative of two experiments. Data are mean ± SEM (n = 3). * *p* < 0.005. NS: not significant.

The variation of intracellular Ca^2+^ in cells can trigger the expression of proteins or activate pathways involved in tumor cell proliferation, invasion and survival [40]. Various Ca^2+^-permeable channels, transporters and pumps were reported to play a key role in these processes [40]. Previously, the inhibition of cleavage of various Furin substrates was reported to affect their ability to mediate calcium mobilization and their biological functions [29,41,42]. To evaluate the effect of Furin repression on the expression of the main Ca^2+^ regulators previously reported to be involved in colon cancer (Figure 7), we directly analyzed their expression in colon cancer cells with or without KRAS or BRAF mutation. These include the plasma membrane Ca^2+^ ATPase (ATP2B4) [43], the purinergic receptor subtype P2RY2A [44] and the calcium channel ORAI1 [45], all upregulated in colon cancer and the calcium voltage-gated channels CACNA1H and CACNA1Cn were found to be downregulated in colon cancer [46].

The repression of Furin in these cells mediated dysregulated expression of the indicated calcium regulators. Some changes were KRAS or BRAF associated and others independently (Figure 7). Those that show significant changes are ATP2B4, which was downregulated in all the Furin-silenced colon cancer cells (Figure 7a–c), and the P2Y receptor, which was specifically repressed in BRAF mutated colon cancer cells (Figure 7d–f). The expression of CACNA1H (Figure 7j–l) and ORAI1 (Figure 7g–i) was downregulated in KRAS-mutated cells, whereas CACNA1C was upregulated in all tested cancer cells (Figure 7m–o). These findings linked Furin activity to several calcium regulators in colon cancer cells with KRAS or BRAF mutation.

## 4. Discussion

To date, the treatment strategies for CRC are mainly based on surgery, radiotherapy, and/or chemotherapy [47]. However, there is still no effective therapeutic approach to prevent the recurrence and metastasis of CRC. In addition, several patients rapidly develop chemoresistance in advanced stages of the disease [48]. Therefore, it is vital to establish new therapeutic drugs or strategies that can effectively eradicate cancer cells and to prevent resistance to treatments that cause tumor relapse. The implication of CSCs in tumor initiation and resistance to therapy has presented new challenges for cancer treatment. To this end, we have identified the implication of the PCs in the malignant phenotype of CSCs. We found that inhibition of the PCs activity in these cells reduces their growth, survival, and invasion. We also identified the implication of the PCs in several markers of stemness and calcium regulators in colon cancer cells with KRAS or BRAF mutation.

The tissue origin of CSCs is still unclear. However, various studies reported that the generation of CSCs seemed to be related to pathways regulating normal stem cells that were altered due to newly occurring mutations, leading to the acquisition of malignant and metastatic phenotypes, such as uncontrolled proliferation, apoptosis escape, and invasiveness [49,50]. Indeed, normal stem cells show a longer lifecycle compared to their differentiated cells, and are thereby more exposed to mutagens and external factors that induce these mutations and subsequently CSCs generation [51]. Well-differentiated cells can also acquire a stem-like phenotype following several mutations involved in self-renewal genes expression. The acquisition of the mesenchymal characteristics by differentiated cells also leads to CSCs phenotype [51,52]. Previously, an association between KRAS mutations and CSC markers expression was reported in CRC patients that induced stemness of CRC cells with APC mutation. KRAS was found to induce sphere formation, chemoresistance, and expression of stem cell markers [7].

Similarly, BRAF mutation was associated with the expression of CSC markers, and advanced cancer stage and metastasis, and BRAF inhibitors induce epithelial re-differentiation in human CRC cell lines [53,54]. The expression of CSC markers in the presence of BRAF mutation was linked to c-MYC and HIF-1α, which are downstream molecules of the mitogen-activated protein kinase pathway [55]. In our study, we found that the reduced ability of colon cancer with inhibited PCs activity to mediate tumor growth was associated with reduced expression of the CSC markers LGR5 and NANOG. Interestingly, specific repression of Furin in colon tumors induced by cancer cells with KRAS or BRAF mutation inhibited the expression of Nanog and LGR5 compared to wild-type KRAS and BRAF developed tumors. This is consistent with the previously reported study concerning inhibition of tumor growth by Furin silencing in KRAS or BRAF mutated cells but not in wild-type KRAS and BRAF cells [34].

Recent studies have shown that intracellular calcium mobilization is also important in stem cells’ capacity for self-renewal, proliferation, and differentiation. Indeed, the expression of a wide variety of calcium regulators and channels was reported for various stem cell types [56,57,58], suggesting that altered expression of these regulators and channels in stem cells may participate in CSCs malignant phenotype and could also represent important targets against CSCs. Previously, we revealed the implication of the PCs activity in the regulation of calcium mobilization in cancer cells [29,32,41] and the requirement of several PCs substrates cleavage by PCs in the mediation of calcium-related cellular function as cell proliferation, survival, and migration [29,41]. In this study, the analysis of the expression of various calcium regulators in colon cancer with repressed Furin revealed their dysregulated expression. These changes were KRAS or BRAF mutation-dependent and others independent. For example, the expression of ATP2B4, a Plasma membrane Ca^2+^ ATPases (PMCAs) known as the major ATP-consuming pumps responsible for Ca^2+^ extrusion from the cells, is repressed in colon cancer with mutated or WT KRAS or BRAF. In contrast, expression of P2RY2A, a purinergic receptor subtype involved in the mobilization of cytosolic Ca^2+^ [59] and implicated in numerous cancer hallmarks [60,61] was downregulated only in BRAF muted cancer cells. P2Y2-mediated intracellular Ca^2+^ increases have been implicated in the proliferation and migration of hepatocellular carcinoma cells in mice [59] and the migration of ovarian carcinoma cells [62]. The reasons for this divergence involving KRAS and BRAF mutations in cancer cells lacking Furin are not presently clear, but several mechanisms may be postulated. Differences in KRAS and BRAF to mediate separated pathways in the absence of Furin may be a contributing factor. Indeed, although KRAS and BRAF both activate the MAPK pathway, they also show pathway divergence, such as the ability of KRAS to activate more signal pathways compared to BRAF [63], and compared to BRAF, KRAS is involved in its feedback control [64]. Furthermore, KRAS- and BRAF-mutated tumors have distinct molecular features and therapeutic profiles compared with non-mutated tumors [65].

## 5. Conclusions

This study examined the importance of PCs in the malignant phenotype regulation of CSCs and identified the implication of the impact of KRAS and BRAF mutations on the effect of Furin repression in these cells. The study also identified dysregulated expression of several calcium regulators in cancer cells with these mutations lacking Furin activity. Taken together with the previous studies [31,32,33,34], the present findings suggest that the ability of reagents that interfere with PCs, particularly Furin, could potentially have therapeutic effects by regulating calcium regulators in colon cancer with KRAS or BRAF mutation.

## Data Availability

The data presented in this study are available on request from the corresponding author.

## Supplementary Materials

The following supporting information can be downloaded at https://www.mdpi.com/article/10.3390/cancers14051195/s1, Table S1: Primers used in the study.
