# Supplementary material for: Role of Furin in Colon Cancer Stem Cells Malignant Phenotype and Expression of LGR5 and NANOG in KRAS and BRAF-Mutated Colon Tumors
Source: Cancers (Basel). 2022 Feb 25;14(5):1195. doi: 10.3390/cancers14051195 (PMC8909039; doi:10.3390/cancers14051195)
Supplement: Supplementary file 1 [file cancers-14-01195-s001.zip › cancers-1569752-proofed supplementary 2.pdf]

Table S1. Primers used in this study.

Primers	5'->3'
GAPDH human Fwd	TGGACTCCACGACGTACTCA
GAPDH human Rev	AATCCCATCACCATCTTCCA
b-Actin human Fwd	CGTACCACTGGCATCGTGAT
b-Actin human Rev	GTGTTGGCGTACAGGTCTTT
Nanog human Fwd	TGAAGAAAACTATCCATCCTTGCA
Nanog human Rev	GGAGGAAGGAGGAGGAGAGACAGT
Oct4 human Fwd	CCCACACTGCAGCAGATCAG
Oct4 human Rev	CACACTCGGACCACATCCTTCT
Sox2 human Fwd	CTGCGAGCGCTGCACAT
Sox2 human Rev	GAGCGTCTTGGTTTTCCGCCGG
Lgr5 human Fwd	CTCCCAGGTCTGGTGTGTTG
Lgr5 human Rev	GAGGTCTAGGTAGGAGGTGAAG
STIM2 human PrimePCR™ SYBR® Green Assay	qHsaCID0009360
ATP2A3 human PrimePCR™ SYBR® Green Assay	qHsaCID0016670
ATP2B4 human PrimePCR™ SYBR® Green Assay	qHsaCID0010124
ATP2A2 human PrimePCR™ SYBR® Green Assay	qHsaCID0011088
ORAI1 human PrimePCR™ SYBR® Green Assay	qHsaCED0003027
CACNA1C human PrimePCR™ SYBR® Green Assay	qHsaCED0045498
TRPM8 human PrimePCR™ SYBR® Green Assay	qHsaCED0003903
CACNA1H human PrimePCR™ SYBR® Green Assay	qHsaCID0017144
STIM1 human PrimePCR™ SYBR® Green Assay	qHsaCED0004653
P2RX5 human PrimePCR™ SYBR® Green Assay	qHsaCED0004972
P2RX7 human PrimePCR™ SYBR® Green Assay	qHsaCID0012839
P2RY2 human PrimePCR™ SYBR® Green Assay	qHsaCED0002726
P2RY4 human PrimePCR™ SYBR® Green Assay	qHsaCED0019170
GAPDH mouse Fwd	AATCCCATCACCATCTTCCA
GAPDH mouse Rev	CGTCTTCCCCCCTGCAT
b-Actin mouse Fwd	GATTACTGCTCTGGCTCCTAGC

b-Actin mouse Rev	GATTACTGCTCTGGCTCCTAGC
Nanog mouse Fwd	TTCTTGCTTACAAGGGTCTGC
Nanog mouse Rev	AGAGGAAGGGCGAGGAGA
Oct4 mouse Fwd	GTTGGAGAAGGTGGAACCAA
Oct4 mouse Rev	CTCCTTCTGCAGGGCTTTC
Sox2 mouse Fwd	CACAACTCGGAGATCAGCAA
Sox2 mouse Rev	CCTCGGGAAGCGTGTACTTA
Lgr5 mouse Fwd	CGAGCCTTACAGAGCCTGATACC
Lgr5 mouse Rev	GCTGGTCACATTGAGAAGCA
